# Role of mutant p53 and autophagy on precancerous lesions of gastric cancer progression: A review

**DOI:** 10.1097/MD.0000000000050252

**Published:** 2026-08-21

**Authors:** Qiuyue Wang, Yutao Wu, Wenrong Wang

**Affiliations:** a First Clinical Medical College, Yunnan University of Chinese Medicine, Yunnan, Kunming, China; b First Clinical Medical College, Fujian University of Traditional Chinese Medicine, Fujian, Fuzhou, China; c Department of Spleen and Gastroenterology, The Second Affiliated Hospital of Fujian University of Traditional Chinese Medicine, Fujian, Fuzhou, China.

**Keywords:** autophagy, mutant p53, precancerous lesions of gastric cancer, prevention and treatment of gastric cancer

## Abstract

Precancerous lesions of gastric cancer (PLGC) represent a critical stage in the development of gastric cancer. Multiple complex factors working together can increase the risk of cancer progression. Relevant published papers were collected through a search of the PubMed, Google Scholar, and Web of Science databases. Recent studies have shown that mutant p53 can impede processes like cell cycle arrest, cell apoptosis, and DNA damage repair, which promotes the proliferation of cancer cells and influences the transition from PLGC to gastric cancer. Autophagy, a process of lysosomal degradation for abnormal proteins or damaged organelles, exhibits dual roles in the process of carcinogenesis (either promoting cell death or facilitating survival). Based on recent research findings, this article systematically discusses the critical roles of mutant p53 and autophagy in the development of PLGC. It also addresses the cross-regulatory relationship between mutant p53 and autophagy, wherein mutant p53 inhibits autophagy, and the regulation of autophagy, in turn, affects the stability of mutant p53. These insights may provide new theoretical support and potential therapeutic strategies for the early prevention and treatment of gastric cancer.

## 1. Introduction

Gastric cancer is a prevalent cancer of the digestive system which is characterized by a poor prognosis. According to the latest data from Global Cancer Statistics 2020, gastric cancer ranks fifth worldwide in terms of incidence rate and fourth in terms of mortality rate. However, in China, both the incidence and mortality rates are the highest globally, accounting for 49.3% and 58.3% of the total cases, respectively.^[1]^ Precancerous lesions of gastric cancer (PLGC) represent a critical stage in the development of gastric cancer. Following the well-established Correa intestinal gastric cancer development model, it is recognized that gastric mucosa Dysplasia is the most significant PLGC, often arising within the context of chronic atrophic gastritis (CAG) and intestinal metaplasia (IM), with a notably high risk of cancer development.^[2]^ Therefore, the early detection and standardized treatment of PLGC can effectively reduce the likelihood of its progression to gastric cancer and help alleviate the cancer burden in China.

PLGC comprises multiple stages of histopathological changes in the gastric mucosa, and its pathogenesis is rather complex. With the advancement of genomics technology, it has been observed that mutations in oncogenes or their overexpression play a significant role in PLGC development. The oncogene p53, being a common and critical oncogene, exhibits the highest frequency of mutation. Mutant p53 loses its tumor-suppressing function while gaining oncogenic function, thus stimulating cancer cell proliferation, infiltration, and metastasis.^[3]^ Additionally, autophagy, a cellular process involving the self-degradation and recycling of accumulated abnormal proteins and damaged organelles, helps maintain cellular homeostasis and regulates cancer cell survival or apoptosis.^[4]^ Recent studies have shown that there is a complex interplay and regulatory relationship between mutant p53 and autophagy, both participating in multiple cellular biological processes. Moreover, the overexpression of mutant p53 and the inhibition of autophagy contribute to PLGC progression. While both mutant p53 and dysregulated autophagy are implicated in PLGC, their intricate interplay and its therapeutic implications have not been comprehensively reviewed. Hence, this study aims to comprehensively explore the interaction between mutant p53 and autophagy during PLGC progression, offering novel theoretical insights and potential therapeutic approaches for the prevention and treatment of early gastric cancer.

We systematically searched electronic databases including PubMed, Google Scholar, and Web of Science, and used combinations of keywords such as “mutant p53,” “Autophagy,” “PLGC,” “Prevention and Treatment of Gastric Cancer,” “Regulatory mechanism,” and “therapeutic targets.” The search was conducted until April 2025. Studies were included if they focused on mutant p53, autophagy regulation, or the interaction between mutant p53 and autophagy in the progression of PLGC, and both original research and review articles were considered. Meanwhile, studies focusing exclusively on non-PLGC-related diseases or those unrelated to mutant p53 or autophagy regulation were excluded. The researchers collated relevant literature to complete this review.

Ethical Statement: This study is a narrative review article aimed at synthesizing and analyzing findings from previously published literature. No new data involving human subjects or animals were collected for this study; thus, ethical approval from an institutional review board and informed consent were not required.

## 2. The mutant p53 protein

As a tumor suppressor protein, p53 is encoded by the Tp53 gene, containing 393 amino acid residues and a complex structural domain.^[5]^ Normally, p53 protein levels are kept low in the cytoplasm and degraded by MDM2, an E3 ubiquitin ligase with ligase activity.^[6]^ In response to stress signals like hypoxia, DNA damage, or reactive oxygen species (ROS), different protein mediators transmit stress signals to the p53 protein and activate it, resulting in increased concentration and activity. P53 binds to target genes with p53 DNA sequence elements, triggering gene transcription and regulating signaling pathways such as cell apoptosis or senescence, cell cycle arrest, and DNA damage repair. This helps in tumor suppression and maintaining homeostasis. It can also establish positive or negative feedback loops with adjacent cell signaling pathways to regulate p53 activity.^[7]^ However, p53 is most prone to mutation in cancer. The mutant p53 protein loses its oncogenic function by binding to proliferative or antiapoptotic target genes, thus playing a critical role in cancer development.^[7,8]^

The mutant p53 protein is closely linked to mutations in the Tp53 gene, located on the short arm of chromosome 17, resulting in alterations to the p53 protein because of chromosomal deletions. These changes cause a prolonged half-life and increased expression levels of the p53 protein in cells or tissues, thereby producing a mutated p53 protein.^[9]^ The Tp53 mutations primarily involve missense mutations formed by the substitution of a single amino acid, and the central DNA binding domain plays a crucial role in p53 mutations. Mutation sites in p53 can alter the amino acid residues binding to DNA sequences of target genes, directly impacting the structure of the p53 protein and diminishing its ability to bind to DNA.^[10]^ Mutations in the p53 protein confer oncogenic activity, which can manifest as loss of function, gain of function (GOF), or dominant negative effect.^[11]^

MDM2, as a specific ubiquitin ligase for the p53 protein, negatively regulates p53 expression levels. Meanwhile, MDM2 also serves as a downstream target gene directly regulated by p53. Therefore, activation of p53 promotes MDM2 transcription, while the increase in MDM2 levels in turn accelerates the degradation of the p53 protein. The interaction between MDM2 and p53 establishes a negative feedback regulatory system, maintaining the balance of p53 protein in normal cells.^[12]^ However, mutations in p53 occurring during carcinogenesis induce conformational changes that disrupt its normal binding to MDM2. This disruption weakens or even eliminates MDM2’s ability to degrade the mutant p53 protein, resulting in stable high expression and abnormal accumulation of mutant p53 in cancer cells.^[11]^ Additionally, mutant p53 also fails to effectively trigger the normal transcription of MDM2, as its core domain mutation exhibits a strong binding affinity with MDM2’s acidic domain, hindering MDM2’s ubiquitin ligase degradative function.^[13]^ Furthermore, various posttranslational modifications of proteins regulate the p53 protein. Increased levels of phosphorylation or acetylation can further stabilize and enhance the oncogenic activity of mutant p53, preventing its degradation by MDM2 and boosting its transcriptional capacity and GOF effect.^[14]^

Heat shock protein 90 (HSP90), functioning as a molecular chaperone protein, also plays a vital role in maintaining the stability of mutant p53. Histone deacetylase 6 (HDAC6) is highly expressed in cancer, which not only participates in the acetylation and deacetylation of histones, targets nonhistone substrates, and regulates the processes of cancer cell proliferation, metastasis, and invasion, but also upregulates tumor-related immune factors and promotes tumourigenesis and progression.^[15]^ HSP90 protein, as one of the nonhistone substrates of HDAC6, may trigger HSP90 deacetylation and augment its expression continually through the HDAC6/Hsp90 complex mechanism.^[16]^ Furthermore, by forming stable complexes with mutant p53, HSP90 can shield mutant p53 from degradation by ubiquitin ligases like MDM2, thus enhancing the accumulation of mutant p53 in tumors and stabilizing its GOF oncogenic activity.^[17]^

## 3. The impact of mutant p53 on PLGC

Mutant p53 proteins serve as a potential marker for cancer detection and diagnosis. In healthy tissues, the expression of p53 is not easily detected. Whereas in most common cancers and precancerous lesions, p53 has a prolonged half-life and is more susceptible to mutation, and therefore high levels of mutant p53 can be detected by immunohistochemical methods and quantitative enzyme-linked immunosorbent assays.^[10]^

The expression of mutant p53 is crucial for the progression of PLGC to gastric cancer, as shown in Table 1. Early studies have shown that the expression of mutant p53 increases with the severity of gastric mucosal lesions. A retrospective study analyzed the morphological and immunohistochemical characteristics of gastric mucosal biopsy specimens, revealing that compared to atypical hyperplasia caused by gastritis, IM, and epithelial injury of the gastric mucosa, the mutant p53 expression rate progressively increased across low-grade and high-grade dysplasia.^[18]^ Tang et al found that the expression of p53 protein and Mdmx significantly increased during the transition from normal gastric mucosa to IM and then to gastric cancer. Moreover, TP53 mutation was a key event in the neoplastic transition from IM to gastric cancer, and the Mdmx-p53 axis promoted the development of gastric cancer.^[19]^ Ming et al showed that the expression rate of mutant p53 protein in early gastric adenocarcinoma tissues was significantly higher than in normal gastric mucosa, and correlated with cellular atypia, serving as an auxiliary indicator for the diagnosis of early gastric cancer.^[20]^ Based on the above studies, it has been shown that genetic instability exists in the early progression of gastric cancer, and p53 gene mutations can promote the development of different pathological stages of PLGC, serving as a marker for detecting PLGC and its progression to gastric cancer. The presence of mutant p53 protein may influence the progression of PLGC by regulating multiple signaling pathways and gene expression. Helicobacter pylori is recognized as a significant factor in the progression from normal gastric mucosa to PLGC malignancy or gastric cancer. Recent studies have revealed that *H. pylori* infection, especially when positive for the cytotoxin-associated gene A (CagA), can trigger genomic instability, resulting in the accumulation of mutated genes. CagA interacts with the protein kinase PAR1b, inhibiting the phosphorylation of the tumor suppressor gene BRCA1 protein and affecting its nuclear translocation, thereby promoting DNA double-strand breaks and damage response in cancer cells. This process of genomic instability contributes to the accumulation of p53 mutations, further stimulating the proliferation of CagA-related lesion cells and their transformation into cancer cells.^[22]^ Meanwhile, mutant p53 enhances the EGFR signaling pathway, regulating its mediated cytogenesis and antiapoptotic activity, thereby promoting abnormal proliferation, differentiation, and apoptosis resistance in gastric mucosal epithelial cells.^[23]^ Furthermore, the expression of mutant p53 is associated with VEGF, which promotes tumor angiogenesis by regulating VEGF and facilitating the induction of carcinogenesis.^[21]^

**Table 1:** Studies on p53 mutation in PLGC.

Research models	Findings	Clinical correlations	References
Human gastric mucosa biopsy specimens	The intensity of TP53 expression correlates with the degree of dysplasia, and a progressive increase in mutation-type pattern expression rates across atypical hyperplasia, low-grade and high-grade dysplasia groups	p53 gene is instrumental in diagnosing gastric atypical hyperplasia and dysplasia.	[18]
Human gastric mucosal tissue with early stage IM and late-stage IM which harbored concurrent GC	A significant increase in p53 protein expression during the transition from normal gastric mucosa to IM and then to GC. The TP53 mutation status varied between IM and GC, and Mdmx expression also significantly increased	TP53 mutations promote the transition from gastric precancerous lesions to gastric cancer, and the Mdmx-p53 axis involves in the progression from IM to GC.	[19]
Human gastric mucosal tissue with early gastric adenocarcinoma	In early gastric adenocarcinoma tissues, the expression rate of mutant p53 protein was significantly higher than in normal gastric mucosa, and closely correlated to cell atypia.	Mutant p53 protein can be used as an auxiliary index for the diagnosis of early gastric cancer.	[20]
Human gastric cancer tissue	Expression of VEGF and P53 was associated with histological type and lymphatic metastasis of gastric cancer	The mutant P53 promotes angiogenesis generation in gastric cancer by adjusting VEGF	[21]

IM = intestinal metaplasia, PLGC = precancerous lesions of gastric cancer.

## 4. Autophagy induction process

Autophagy is a dynamic degradation and recycling process that includes macro-autophagy, micro-autophagy, and chaperone-mediated autophagy. These 3 types of autophagy perform degradation through lysosomes, where the degraded components are continuously reshaped and recycled, providing new energy for renewal processes during cell development and differentiation, and maintaining the balance of normal cells in the body.^[4]^ Autophagy can also phagocytose and eliminate damaged organelles such as misfolded or abnormally aggregated proteins, mitochondria, endoplasmic reticulum, and peroxisomes, as well as intracellular pathogens, thereby playing a role in cellular protection.^[24]^

The process of autophagy requires the participation of multiple proteins. *ATG1* and its homolog *ULK1* are the initial proteins that activate autophagy, regulated by the energy-sensing kinase Mammalian target of Rapamycin (mTOR). mTOR can phosphorylate *ATG1*/*ULK1* to inhibit autophagy. Conversely, inhibiting mTOR can block the phosphorylation of *ATG1*/*ULK1*, thereby activating autophagy.^[25]^ Furthermore, Beclin-1, a marker of autophagy activation, is activated by *ATG1*/*ULK1* to promote autophagy progression. Antiapoptotic proteins, such as Bcl-2, are negatively correlated with autophagy and can bind to Beclin-1 to inhibit autophagic activity.^[26]^ Microtubule-associated protein light chain 3 (LC3) exists in 2 forms, LC3-I and LC3-II. During autophagy, LC3-I converts to LC3-II, playing a role in auto phagosome extension and the formation of autophagic lysosomes, and serving as a crucial marker for serving autophagy levels.^[24]^ P62, a substrate for autophagy and a marker for autophagic inactivation, binds to LC3 (ATG8) and interacts with various ubiquitinated proteins, which are encapsulated by autophagosomes to participate in intracellular substances degradation through autophagy. An increase in the level of P62 indicates that autophagic degradation activity is inhibited.^[27]^

## 5. The role of autophagy in PLGC

Autophagy is an intracellular self-regulatory physiological process. Due to the influence of the cellular microenvironment and the specific stage of the disease, it exerts dual roles in different cells and at different stages of the same cell, playing a significant role in the formation and progression of cancer.^[28]^ PLGC is a critical stage in the “inflammation-cancer” transformation of gastric mucosa, characterized by morphological and structural changes in gastric mucosal cells, the gradual emergence of malignant pathological features, and eventual progression to gastric cancer. The autophagy mechanism plays a distinct regulatory role at different stages of PLGC development.^[29]^ The autophagic mechanism exerts distinct regulatory roles at different stages of PLGC development, as shown in Table 2.

**Table 2:** Studies on autophagic mechanism in PLGC development.

Research models	Findings	Clinical correlations	References
GES-1 cells and CAG rat model induced by MNNG	Activation of the TGF-β1/PI3K/Akt/mTOR pathway and decreased expression of autophagic proteins LC3-II and Beclin-1	Autophagy inhibition is associated with inflammatory damage of gastric mucosa and GES-1 cells, which may promote the progression of CAG	[30,31]
Mongolian gerbil gastric tissue and GES-1 cells with H. Pylori infection and early gastric cancer tissues of human	H. Pylori infection led to upregulation of autophagy substrate p62 and downregulation of repair protein Rad51, DNA repair damage	Chronic H. pylori infection may promote gastric cancer by causing loss of autophagy, inducing Rad51 ubiquitination and inhibiting the ability of DNA damage repair	[32]
GES-1 cells and mouse models with H. Pylori infection	H. Pylori infection downregulated the expression of miR-1298-5p in vitro and in vivo, increased the expression of MAP2K6, and inhibited autophagy	Regulating miR-1298-5p/MAP2K6/p38 MAPK axis to inhibit autophagy may be a new way to treat H. Pylori infection and H. Pylori related GC	[33]
The rat model of PLGC	PI3K/AKT/mTOR signaling pathway activation and p53/AMPK/*ULK1* signaling pathway inhibition, while increasing the formation of autophagosomes and autolysosomes as well as the expression of Beclin-1 and LC-3II	Sustained high levels of autophagy may contribute to PLGC deterioration	[34]
The rat model of PLGC	The expression of autophagy factors Ambra1, Beclin-1, LC3, p62, ATG5 and *ATG1*2 significantly increased, while the expression of apoptosis factors Bcl-XL, Bcl-2/Bax was increased and Caspase-3 decreased	Abnormal activation of autophagy and inhibition of apoptosis may promote the pathological features such as IM and Dys	[35]
The rat and cell model of PLGC	PDCD4 expression decreased, ATG5 expression increased, autophagy level increased	Regulating PDCD4-ATG5 signaling pathway to inhibit autophagy can improve gastric mucosal damage and inhibit PLGC cell proliferation	[36]

PLGC = precancerous lesions of gastric cancer.

On the one hand, during the early stage of PLGC, moderate autophagy clears pro-oncogenic or lesioned proteins and organelles, maintains intracellular homeostasis, suppresses inflammatory progression, and prevents the accumulation of genetic damage and even malignant transformation, thereby inhibiting the carcinogenesis of gastric mucosal cells.^[37]^ Tong et al found that decreased expression levels of autophagy activation-related proteins LC3-II and Beclin-1 were associated with inflammatory responses and pathological damage in gastric mucosal epithelial cells and tissues of CAG, and Traditional Chinese medicine (TCM) could alleviate gastric mucosal inflammatory injury and improve CAG by inducing autophagy.^[30,31]^ Autophagy induction serves as a defence mechanism of cells against intracellular pathogenic micro-organisms. *H. pylori*, a key etiological factor in CAG and PLGC, expresses 2 virulence factors: CagA and vacuolating cytotoxin A, which could promote *H. pylori* colonization in the gastric mucosa and the accumulation of toxic products by inhibiting autophagy pathway activation and autolysosome formation, leading to various malignant processes such as inflammatory responses, oxidative stress, and enhanced glycolytic activity in gastric mucosal tissues.^[38]^ Persistent *H. pylori* infection resulted in loss of autophagy and accumulation of the autophagy substrate p62, induced ubiquitination of the DNA repair protein Rad51, promoted *H. pylori*-induced DNA damage, and accelerated the carcinogenesis of gastric mucosal epithelial cells.^[32]^ Furthermore, *H. pylori* infection downregulated the expression level of miR-1298-5p, which inhibited autophagy through activation of the MAP2K6/p38 MAPK axis, thereby promoting the proliferation and migration of gastric mucosal carcinogenic cells.^[33]^

On the other hand, when gastric mucosal lesions accumulate to a certain extent, the complex local microenvironment including hypoxia, ischemia, persistent inflammatory states, or dysregulation of signaling pathways in PLGC, could lead to abnormally increased autophagy, and it not only facilitated the repair of damaged organelles in dysplastic cells within PLGC and promoted their survival, but also exacerbated injury and death in normal gastric mucosal cells.^[37]^ Zhang et al observed that a significant increase in the number of autophagosomes, autophagic vesicles, and autolysosomes in the gastric mucosal tissues of PLGC rats compared with the normal control group, along with elevated expression levels of Beclin-1 and LC3-II, which indicated that sustained high levels of autophagy could support the survival of IM and Dysplasia cells and damage normal gastric mucosal cells, thereby further promoting the progression of PLGC.^[34]^ Cai et al found that under long-term MNNG stimulation, the expression of autophagy-related factors including Ambra1, Beclin-1, LC3, p62, ATG5 and *ATG1*2 was significantly upregulated in the gastric mucosal tissues of PLGC rats, and the antiapoptotic factors Bcl-XL and Bcl-2/Bax expression increased, while Caspase-3 expression decreased; the abnormal activation of autophagy coupled with inhibition of apoptosis might facilitate the progression of PLGC.^[35]^ Zhu et al demonstrated that inhibiting autophagy via regulation of the PDCD4-ATG5 signaling pathway could ameliorate gastric mucosal damage and suppress proliferation in a PLGC rat model.^[36]^ Therefore, regulating autophagy is of great significance for effectively reversing PLGC.

## 6. Cross-regulation of mutant p53 and autophagy

There is a complex cross-regulatory relationship between mutant p53 and autophagy. Mutant p53 protein might impact the cellular autophagy process by disrupting the normal regulation of the autophagy signaling pathway. Conversely, autophagy can also regulate the stability and function of mutant p53 protein, affecting its oncogenic activity.

### 6.1. Inhibition of autophagy by mutant p53

**Figure 1.**
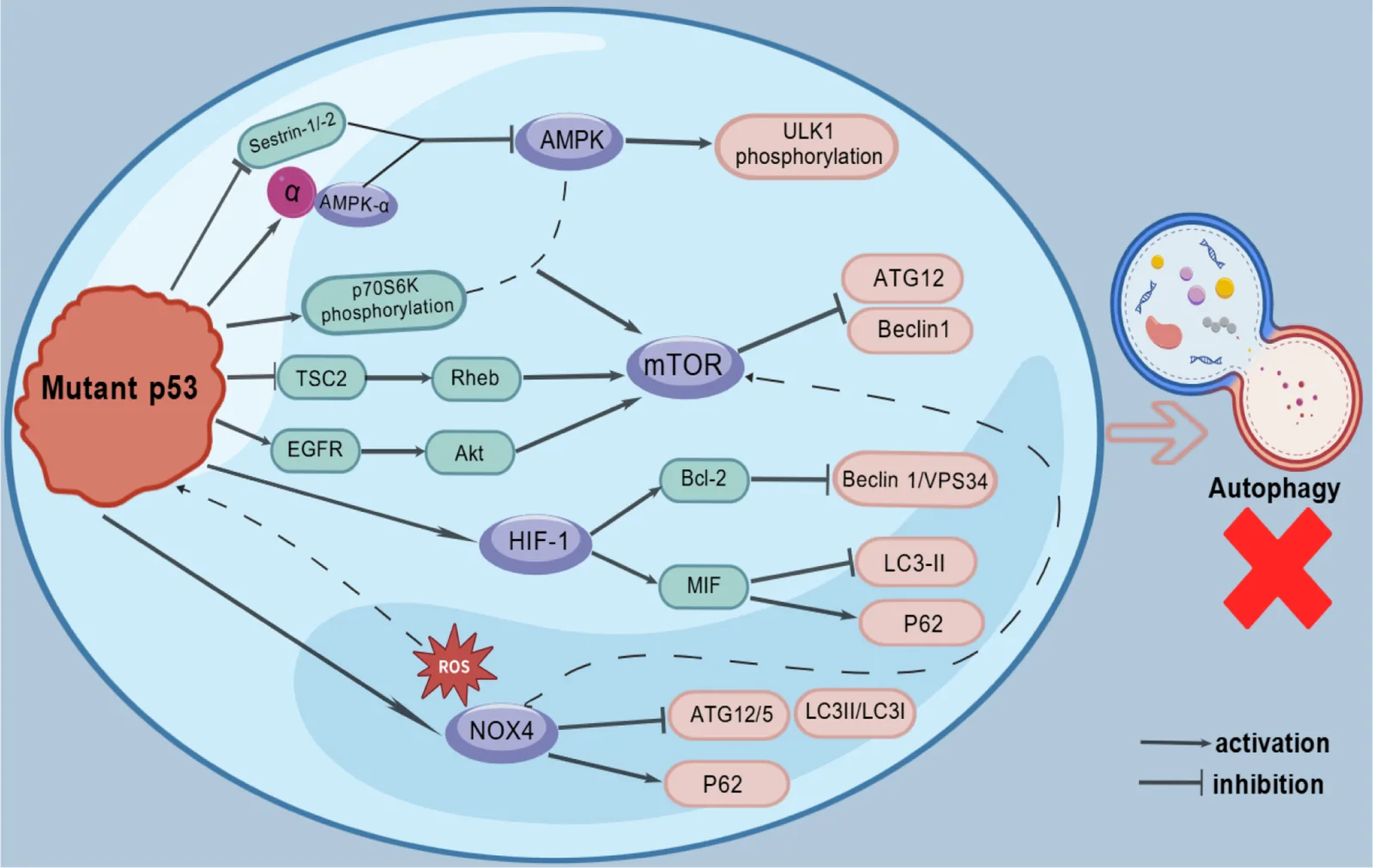
Inhibition of autophagy by mutant p53. AMPK = AMP-activated protein kinase, HIF-1 = hypoxia inducible factor-1 alpha, mTOR = mammalian target of rapamycin, NOX4 = NADPH oxidase 4.

Some autophagy genes, such as *Atg2*, *Atg4*, *Atg7*, *Atg10* and *ULK1*, can function as direct target proteins of wild-type p53. p53 binds to these autophagic target proteins and activates their function, promoting autophagy. Additionally, p53 responds to DNA damage-induced autophagy, which promotes apoptosis of pathological cells and inhibits their differentiation.^[39]^ However, p53 has a dual role in regulating autophagy, depending on its localization in the cell. Unlike wild-type p53, which is located in the nucleus, mutant p53 is often located in the cytoplasm and regulates autophagy in the opposite way.^[40]^ It was found that cytoplasmic p53 significantly inhibits autophagy, and this inhibitory effect persists in nucleated cells.^[40]^ Furthermore, mutant p53 can inhibit autophagic activity by affecting autophagy-related signaling pathways or factors, exerting its GOF oncogenic role. As shown in Figure 1.

#### 6.1.1. The AMP-activated protein kinase (AMPK) signaling pathway

AMPK, a cellular energy stress sensor, interacts with rapamycin target (mTOR) to regulate cell growth.^[41]^ Research indicates that wild-type p53 can directly activate the phosphorylation of autophagy kinase *ULK1*-related sites by regulating AMPK, increasing the expression of the autophagic markers Beclin-1 and LC3-II, and promoting autophagic activation.^[34]^ In contrast, mutant p53 interacts with the AMPK-α subunit, directly inhibiting AMPK activation and thereby blocking its activation of the autophagy kinase *ULK1*.^[42]^ Moreover, mutant p53 indirectly inhibits AMPK activity by suppressing its activators Sestrin-1 and Sestrin-2, and blocking autophagy occurrence.^[43]^

#### 6.1.2. The Mammalian target of Rapamycin (mTOR) signaling pathway

mTOR is a key factor in regulating the initiation of autophagy and exists in the form of 2 complexes, mTORC1 and mTORC2. Cordani et al revealed that mutant p53 can enhance mTOR signaling by inhibiting AMPK phosphorylation and increasing p70S6K phosphorylation, which inhibits the formation of autophagosomes in cells and their fusion with lysosomes, as well as suppressing the expression of key autophagy genes *ATG1*2 and Beclin-1, ultimately leading to cancer cell proliferation.^[43]^ The mTOR activity is influenced by the TSC complex on the lysosomal enzyme membrane surface and the Rheb protein. Agarwal et al found that mutant p53 can decrease TSC2 expression, inducing the dissociation of the TSC complex from lysosomes and promoting Rheb protein-dependent mTORC1 activation, resulting in weakened autophagy.^[44]^ Tan et al reported a positive correlation between high expression of mutant p53 variants and strong phosphorylation of Akt, which can inhibit apoptosis and autophagy in cancer cells by activating the PI3K/Akt/mTOR classical autophagy pathway.^[45]^ Additionally, several growth factor receptors, such as epidermal growth factor receptor, can participate in the activation of the Akt/mTOR signaling pathway. Muller et al discovered that in cells expressing mutant p53, the phosphorylation level of the epidermal growth factor receptor significantly increases, leading to the activation of downstream Akt/mTOR signaling and mediating autophagy suppression, thereby enhancing the invasive and proliferative capacity of mutant p53-driven cancer cells.^[46]^

#### 6.1.3. Hypoxia inducible factor-1 alpha (HIF-1)

Mutant p53 was discovered to trigger the activation of HIF-1, affecting the tumor microenvironment and synergistically promoting tumor progression.^[47]^ Under hypoxic conditions, the overexpression of HIF-1 can increase the expression of the antiapoptotic protein Bcl-2, promote the binding of Bcl-2 with Beclin-1, and hinder the formation of the Beclin-1/VPS34 complex, thereby inhibiting autophagy.^[48]^ Furthermore, macrophage migration inhibitory factor (MIF) serves as a downstream target of HIF-1 under hypoxia, as well as a strong inhibitor of autophagic cell death. It was found that the increased expression of HIF-1 could activate MIF expression, further reducing the expression level of LC3-II and increasing the expression level of P62, thereby inhibiting autophagic cancer cell death and promoting cancer cell proliferation.^[49,50]^ Therefore, mutant p53 can boost the expression and function of HIF-1, resulting in the inhibition of autophagy by influencing cell apoptosis or the activity of its downstream target gene MIF.

#### 6.1.4. Oxidoreductase NADPH oxidase 4 (NOX4)

The NOX4 protein is the catalytic subunit of the NADPH oxidase complex, which catalyzes molecular oxygen as a source of ROS generation. It does not require external signals or regulatory proteins to exert its transcriptional activity and can quickly respond to changes in oxygen concentration.^[51]^ Mutant p53 can enhance Nox4 expression, thereby promoting ROS generation and leading to the migration and invasion of malignant epithelial cells.^[52]^ The GOF of mutant p53 is also regulated by oxidative stress, with increased ROS production in cells favoring the stability of mutant p53.^[53]^ Additionally, there is a connection between Nox4 and autophagy. Increased Nox4 expression leads to a decrease in the expression of autophagy regulators *ATG1*2/5 and LC3II/LC3I, an increase in p62 expression, and subsequently increased levels of inflammatory cytokines TNF-α, IL-6, and TGF-β. Conversely, decreased NOX4 expression increases autophagy and reduces inflammatory cytokine levels, indicating that NOX4 expression can negatively regulate autophagic responses and influence the generation of the tumor inflammatory microenvironment.^[54]^ Therefore, mutant p53 can inhibit autophagy by enhancing NOX4 oxidative signaling and promoting cell carcinogenesis.

### 6.2. Regulating autophagy affects the stability of mutant p53

Mutant p53 maintains its stable expression by evading MDM2 proteasomal degradation, whereas the autophagy-mediated lysosomal degradation pathway can influence the stability of mutant p53. Rodriguez et al found that elevated intracellular glucose levels increased mutant p53 expression, whereas glucose restriction induced deacetylation of the mutant p53 protein, leading to its degradation via autophagy, indicating that autophagy could be an alternative pathway for mutant p53.^[55]^ Choudhury et al further revealed that under conditions of cell growth, inhibiting autophagy or downregulating key autophagy genes such as *ATG1*/*ULK1*, Beclin-1, or ATG5 stabilized mutant p53; conversely, overexpression of Beclin-1 or *ATG1*/*ULK1* led to the depletion of p53 mutants and blocked tumor progression.^[56]^ The degradation of misfolded protein aggregates by autophagy requires the involvement of various ubiquitin-modifying enzymes, with reduced levels of acetylation and increased levels of ubiquitination being crucial for the autophagic degradation of mutant p53.^[55]^ CHIP, a chaperone-dependent E3 ubiquitin ligase, can ubiquitinate and degrade its substrate proteins via the lysosomal pathway. Maan et al demonstrated that under stress conditions induced by hypoxia or starvation, CHIP mediates lysine residue K63-linked polyubiquitination chains, enhancing autophagic degradation of mutant p53 protein by increasing lysosomal enzyme activity and LC3 expression levels.^[57]^ Furthermore, different autophagic pathways are interconnected, which influences the stability of mutant p53. Helin et al reported that under cellular stress from nutrient deprivation, macro-autophagy activity decreases while chaperone-mediated autophagy is activated, promoting Hsc70 recognition and interaction with the mutant p53 protein, leading to its uptake and degradation by lysosomes, which is conducive to inhibiting the proliferation of mutant p53-dependent cancer cells.^[58]^

## 7. Therapeutic implications and future perspectives

The complex cross-regulation between mutant p53 and autophagy offers a promising therapeutic avenue for intervening in PLGC and preventing its progression to gastric cancer.

### 7.1. Targeting mutant p53

Current therapeutic strategies targeting p53 mainly include 2 approaches: one targets wild-type p53 and inhibits the p53/MDM2 complex to prevent its degradation, potentially restoring tumor-suppressive function of p53, including the use of MDM2-p53 inhibitors^[59]^; the other targets mutant p53 and promotes its degradation, involving autophagy modulation and targeting proteasome/lysosome-related pathways.^[60]^

Related studies have also demonstrated that inhibiting the expression of mutant p53 protein is significant for treating and blocking the progression of PLGC. Zhang et al utilized β-glucan from natural sources to load doxorubicin (DOX), constructing 2 types of nanoparticles (DRP-DOXs) for targeted delivery of therapeutic agents, and the results of an in vitro experimental study indicated that DRP-DOXs effectively alleviated gastric mucosal injury by downregulating oxidative stress and inflammation in gastric mucosal epithelial cells through modulation of the p53 and PI3K pathways, thereby alleviating PLGC.^[61]^ Curcuma zedoaria has been shown to reduce the expression of cancer markers mutant P53 and CD133 in the MNU-induced PLGC mouse gastric tissues by suppressing the Hippo signaling pathway and the expression of LncRNA AFAP1-AS1, thereby inhibiting the malignant progression of PLGC.^[62]^ The results of an animal experiment demonstrated that ginsenoside GRg3 can reduce TP53-induced glycolysis and the expression of the apoptosis regulator TIGAR, induce apoptosis, and inhibit abnormal proliferation and differentiation of gastric mucosal epithelial cells, thereby delaying the progression of PLGC to GC.^[63]^ Qilianshupi decoction is a TCM that is commonly used to treat CAG. A randomized controlled trial in rats with precancerous CAG lesions showed that the components of Qilianshupi prevent PLGC through decreasing the expression of mutant p53 and surviving and regulating telomerase activity and telomere length in CAG.^[64]^

### 7.2. Regulation of autophagy

The autophagy mechanism is closely associated with tumorigenesis and development, playing a dual role in different types and stages of tumors. Due to this dual nature of autophagy, various therapeutic strategies exist for cancer treatment, including the use of autophagy inducers or autophagy inhibitors. Dysregulated autophagy can lead to increased DNA damage, chronic inflammation, and carcinogenesis. Autophagy inducers such as mTOR inhibitors, SIRT1 agonists, and natural products including alkaloids and terpenoids could act synergistically to inhibit cancer cell growth.^[65]^ In vitro experiments found that methylxanthine derivatives (such as caffeine and theophylline) promoted apoptosis and autophagy in gastric cancer cells by activating the tumor suppressor PTEN and inhibiting the PI3K/Akt/mTOR pathway.^[66]^ As a natural active ingredient of Rhodiola rosea, in vitro cellular experiments revealed that its active ingredient Salidroside can inhibit the PI3K/Akt/mTOR pathway, induce protective autophagy and cancer cell apoptosis, ultimately inhibiting the growth of gastric cancer cells.^[67]^

In pro-tumorigenic or tumor cells, autophagy often supports cancer cell survival under complex stress conditions by providing energy, thereby influencing cancer progression and drug resistance. Recent studies have found that impaired autophagic flux caused by disruption of the fusion and degradation processes between autophagosomes and lysosomes could ultimately lead to cancer cell death without promoting their growth.^[68]^ Autophagy inhibitors can be used alone or in combination with anticancer drugs for cancer treatment. In a successful experiment modeling gastric cancer in mice infected with *H. pylori*, findings indicated that using chloroquine alongside *H. pylori* eradication therapy could inhibit gastric cancer development by inhibiting Beclin-1 and LC3B-II protein expression, reducing inflammatory cytokine levels of IL-1β, IL-6, and TNF-α.^[69]^ The in vitro cellular study revealed that chloroquine inhibits autophagy, which enhances the proliferation-inhibiting and apoptosis-inducing effects of cisplatin on gastric cancer SGC-7901 cells. Thus, it provides a rationale for the administration of cisplatin combined with chloroquine in the treatment of gastric cancer patients.^[70]^ A preclinical model of gastric cancer cells with amplified receptor tyrosine kinase Met showed that hydroxychloroquine combined with Met tyrosine kinase inhibitors blocked autophagy flux, thereby inhibiting the survival and growth of gastric cancer cells and enhancing the in vitro anticancer efficacy of Met-TKIs.^[71]^ Timosaponin AIII, a steroidal saponin extracted from the plant Anemarrhena asphodeloides, serves as a novel late-stage autophagy inhibitor. Research utilizing the model of gastric cancer cells and nude mouse subcutaneous tumors revealed that Tim AIII treatment inhibits gastric cancer progression by inhibiting the Keap1-Nrf2 and PI3K/AKT/mTOR pathways while activating the AMPK pathway, thereby inducing oxidative stress and blocking the autophagy flux.^[72]^ In recent years, nanomedicines have shown great potential in cancer diagnosis and treatment due to their enhanced permeability and retention effect, and nanomaterials could also be conjugated with targeting agents, enabling the selective delivery of anticancer drugs to tumors.^[73]^ Song et al established gastric cancer models through in vitro cell and in vivo animal experiments; the results confirmed that nanoparticles loaded with WSGC peptide could inhibit cancer cell proliferation and migration, and increase apoptosis by targeting the Notch signaling pathway and suppressing mitophagy, thereby alleviating chemoresistance in gastric adenocarcinoma.^[74]^ However, current research on autophagy modulators for gastric cancer treatment remains largely focused on preclinical studies, with a lack of relevant clinical trials. Additionally, therapeutics for autophagy regulation also face potential challenges and side effects. For example, the dual regulatory role of autophagy in either promoting or suppressing tumors presents challenges for context-specific targeting, including the selection of indications and the timing of intervention. Then, the toxicity of autophagy inhibitors, including rapid clearance, poor accumulation at tumor sites, and high-dose side effects, limits their therapeutic application in cancer.^[73]^ Meanwhile, the targeted research of novel autophagy inhibitors in patients remains preliminary, and whether they can enhance the efficacy of standard cancer treatments requires further clinical validation.^[75]^

### 7.3. The dual-regulation therapy

TCM has shown favorable therapeutic efficacy in the treatment of PLGC due to its multi-target and multi-component advantages. Through the PLGC model construction in rats, the results showed that Xiao Jianzhong Decoction could alleviate gastric mucosal autophagy and glycolysis in PLGC rats by activating the PI3K/AKT/mTOR pathway and inhibiting the p53/AMPK/*ULK1* pathway, thereby preventing the progression of PLGC.^[34]^ Preclinical studies indicated that the plant-derived saponin compound astragaloside AS-IV effectively protected PLGC rats from gastric mucosal injury by downregulating mutant p53 protein levels, reducing expression of autophagy-related genes Ambra1, Beclin-1, ATG5, LC3, and p62, and inducing apoptosis.^[35]^ Research in vitro and in a patient-derived xenograft ovarian cancer model in vivo confirmed that a novel nanorobotic technology enhances the autophagic degradation of mutant p53 by preparing nanoreceptors that mimic selective autophagy receptors and simultaneously recognize mutant p53 for transport into autophagosomes, thereby demonstrating superior antitumor efficacy.^[76]^ This study also suggests a potential future direction for the treatment of PLGC and gastric cancer.

## 8. Limitations

This review has several limitations that should be noted. Firstly, as a narrative review, this study focuses on synthesizing theoretical advances and complex mechanisms. The included literature may be affected by subjectivity and inherently carries selection bias, without employing systematic or meta-analytic methods that could have enabled quantitative synthesis or minimized potential bias. Secondly, the exploration of mechanisms underlying PLGC development has largely relied on in vitro and animal model studies. While these investigations provide foundational knowledge, clinical trials directly validating these findings in human PLGC tissues remain relatively limited. Additionally, research on the interaction between mutant p53 and autophagy in influencing PLGC remains scarce, and further studies are urgently needed to elucidate the specific regulatory mechanisms and influencing factors of mutant p53 and autophagy in PLGC progression. Finally, the specific regulatory mechanisms of autophagy make it a promising approach for comprehensive treatment of various clinical diseases. However, due to the influence of multiple complex factors such as tumor cell types, microenvironments, and specific disease stages, autophagy exhibits high complexity and context-dependence, posing significant challenges to targeting autophagy regulation as a precision therapy.

## 9. Conclusion

The p53 protein mutation and the dual activities of autophagy (promoting cell death or survival) are closely associated with disease carcinogenesis. Therefore, targeting mutant p53 and regulating autophagy are highly important for the prevention and treatment of tumor development. This review illustrates that mutant p53 and autophagy play critical roles in PLGC and exhibit a mutual regulatory role. Mutant p53 possesses GOF activity, facilitating cancer cell survival, proliferation, and invasion, thereby promoting the progression of PLGC. Meanwhile, mutant p53 can inhibit autophagy by regulating various transcription factors or signaling pathways, reducing the degradation of mutant p53 by autolysosomes. However, during the early stages of tumor formation, pro-oncogenic cells may be eliminated through the autophagy pathway. When the lesions accumulate to a certain extent, forming a complex state of stress, tumor cells may still evade apoptosis via autophagy and facilitate p53 mutation. Thus, further exploration of the interaction between mutant p53 and autophagy is essential for developing novel strategies for the early prevention and treatment of gastric cancer. Building on this synthesis, we propose that targeting the mutant p53-autophagy axis, particularly using dual-regulation strategies such as those emerging from TCM and nanomedicine, represents a promising frontier for the development of novel interventions to halt the progression of PLGC.

## Author contributions

**Visualization:** Qiuyue Wang.

**Conceptualization:** Wenrong Wang.

**Funding acquisition:** Wenrong Wang.

**Writing – original draft:** Qiuyue Wang.

**Writing – review & editing:** Qiuyue Wang, Yutao Wu.
